# Stable Electrospinning of Core-Functionalized Coaxial Fibers Enabled by the Minimum-Energy Interface Given by Partial Core–Sheath Miscibility

**DOI:** 10.1021/acs.langmuir.1c01824

**Published:** 2021-11-04

**Authors:** Shameek Vats, Manos Anyfantakis, Lawrence W. Honaker, Francesco Basoli, Jan P. F. Lagerwall

**Affiliations:** † Experimental Soft Matter Physics Group, University of Luxembourg, L-1511 Luxembourg, Luxembourg; ‡ Laboratory of Physical Chemistry and Soft Matter, Wageningen University & Research, 6703 DE Wageningen, The Netherlands; § Department of Engineering, Università Campus Bio-Medico di Roma, 00128 Rome, Italy

## Abstract

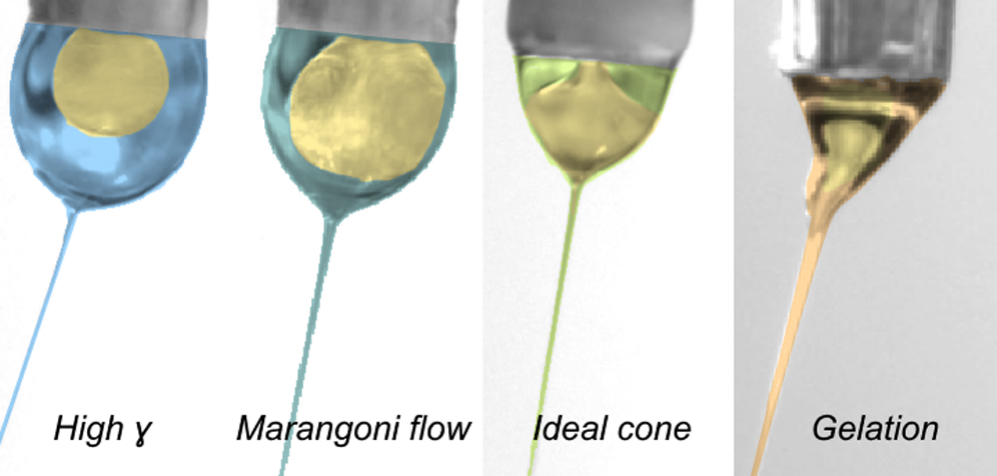

Core–sheath electrospinning is a powerful tool for producing composite fibers with one or multiple encapsulated functional materials, but many material combinations are difficult or even impossible to spin together. We show that the key to success is to ensure a well-defined core–sheath interface while also maintaining a constant and minimal interfacial energy across this interface. Using a thermotropic liquid crystal as a model functional core and polyacrylic acid or styrene-butadiene-styrene block copolymer as a sheath polymer, we study the effects of using water, ethanol, or tetrahydrofuran as polymer solvent. We find that the ideal core and sheath materials are partially miscible, with their phase diagram exhibiting an inner miscibility gap. Complete immiscibility yields a relatively high interfacial tension that causes core breakup, even preventing the core from entering the fiber-producing jet, whereas the lack of a well-defined interface in the case of complete miscibility eliminates the core–sheath morphology, and it turns the core into a coagulation bath for the sheath solution, causing premature gelation in the Taylor cone. Moreover, to minimize Marangoni flows in the Taylor cone due to local interfacial tension variations, a small amount of the sheath solvent should be added to the core prior to spinning. Our findings resolve a long-standing confusion regarding guidelines for selecting core and sheath fluids in core–sheath electrospinning. These discoveries can be applied to many other material combinations than those studied here, enabling new functional composites of large interest and application potential.

## Introduction

Although the idea of making fibers by electrospinning is approaching its centennial anniversary,^1^ it has only been in the last two decades that the technique has truly flourished.^2−8^ The introduction of core–sheath electrospinning using nested capillary spinnerets, often coaxial, has led to an explosion of creativity, with a diversity of functional nano- and microfibers with a variety of internal morphologies being successfully electrospun.^9−13^ Pioneering contributions in demonstrating the potential of dual-phase coaxial electrospinning for making controlled core–sheath fibers were published by Sun et al.,^14^ Yu et al.,^15^ and Li and Xia.^16^ The latter was the first paper to spin coaxial fibers where the core was a nonpolymeric and nonvolatile liquid, thus defining a cylindrical core that could easily be removed after spinning to make hollow tubes. The authors emphasized that the core and sheath liquids must be immiscible for reliable results. This contrasted with the results of Sun et al. and Yu et al., which both were obtained with miscible core and sheath liquids.

While the mineral oil used as core liquid by Li and Xia was largely a sacrificial fluid, its presence ensuring tube-like fiber morphology, several subsequent electrospinning studies incorporated more precious liquids, e.g., phase change materials,^17−19^ liquid crystals (LC),^20−36^ and shear thickening fluids,^37^ to remain as a functional core inside the fibers. These specially selected core liquids enhance the composite fibers with dynamic and responsive performance that the sheath polymer itself is incapable of, while the coaxial fiber geometry provides a powerful means of encapsulating the liquids—which are unspinnable on their own—in a flexible form factor with high surface-to-volume ratio. Several modifications of the fundamental core–sheath electrospinning process have been explored, such as triple-phase coaxial electrospinning^a^, enabled by adding a third nested capillary, which can yield fibers with an intermediate layer between the innermost core and the outermost sheath.^38−41^ With noncoaxial electrospinning using multiple bundled capillaries inside an outer capillary that flows the sheath solution, fibers were produced with multiple core channels, consisting of identical^42^ or different^18,27^ materials. Core–sheath fibers were also obtained using single-phase electrospinning, relying on radial phase separation during spinning.^23,28,32,35,36^ Using the different variations of core–sheath fiber electrospinning, functional composite fibers have been produced for a variety of application scenarios, such as sustained release of drugs,^11,40,43−52^ growth factor,^53,54^ genes,^54,55^ or live cells;^56−58^ enhanced thermal insulation;^17−19^ sensing of volatile organic compounds;^25,29,32,59^ generation of wavy polymer structures;^60^ or sound damping.^37^

Despite the strong interest in core–sheath electrospinning, the answer to the critical question of whether the core and sheath liquids should be miscible or not remains elusive. The original confusion remains, with different teams publishing conflicting views on the matter, both for core–sheath electrospinning and for the closely related challenge of core–sheath electrospray. Several papers reported on spinning cores and sheaths that are fully miscible,^14,15,37,41,43−46,49,53,58,61,62^ some emphasizing the importance of low interfacial tension, γ_cs_, between the two liquids.^15,63^ Others have maintained that cores and sheaths should be immiscible,^16−18,30,42,55,64−66^ often referring to the original Li and Xia work, which even showed evidence of loss of core–sheath structure when miscible fluids were spun.^16^ In our own research, we have encountered problems with both approaches, frequently failing to produce core–sheath fibers either due to excessive γ_cs_ between immiscible liquids or due to miscible liquids without a well-defined interface fusing together, leaving no core–sheath structure in the produced fibers. Given the scarcity of publications of negative results, we believe other teams may have faced similar issues without reporting them.

The purpose of this paper is to clarify the situation by conducting a thorough and systematic investigation of core–sheath interfacial phenomena and how they are affected by (im-)miscibility between the two liquids, using a liquid crystal (LC) mixture as a model fiber-functionalizing core fluid that is nonvolatile and nonpolymeric, and three representative polymer solutions for the sheath. We focus particularly on the quality of the Taylor cone, of fundamental importance to the success of any electrospinning process, since the jet that will form the fiber emanates from the Taylor cone apex. We recently demonstrated that humidity in the spinning environment can ruin the quality and stability of the Taylor cone and that certain core fluids during coaxial electrospinning can amplify this sensitivity to humidity.^67^ We now move the attention from the Taylor cone outside to the core–sheath interface, where we find that neither complete miscibility nor complete immiscibility is advisable: the former triggers sheath gelation and loss of core–sheath structure; the latter gives rise to core breakup in the jet, often already in the Taylor cone. The ideal is partial miscibility with a miscibility gap creating a distinct core–sheath interface, yet its interfacial tension γ_cs_ is much reduced since the two bounding phases contain the same constituents, only at different compositions. The low γ_cs_ allows an uninterrupted core flow from the inner spinneret needle to the Taylor cone apex, where it enters the jet that forms the fiber, and it also prevents the Rayleigh–Plateau instability from breaking up the continuous core within the jet. We also believe that solutal Marangoni flow, to the best of our knowledge not previously discussed in the context of core–sheath electrospinning, can have a highly disruptive influence, and we find that the problem can be avoided by premixing a small fraction of sheath solvent into the core prior to electrospinning.

## Experimental Section

### Polymer Solutions and Liquid Crystals

**Figure 1:**
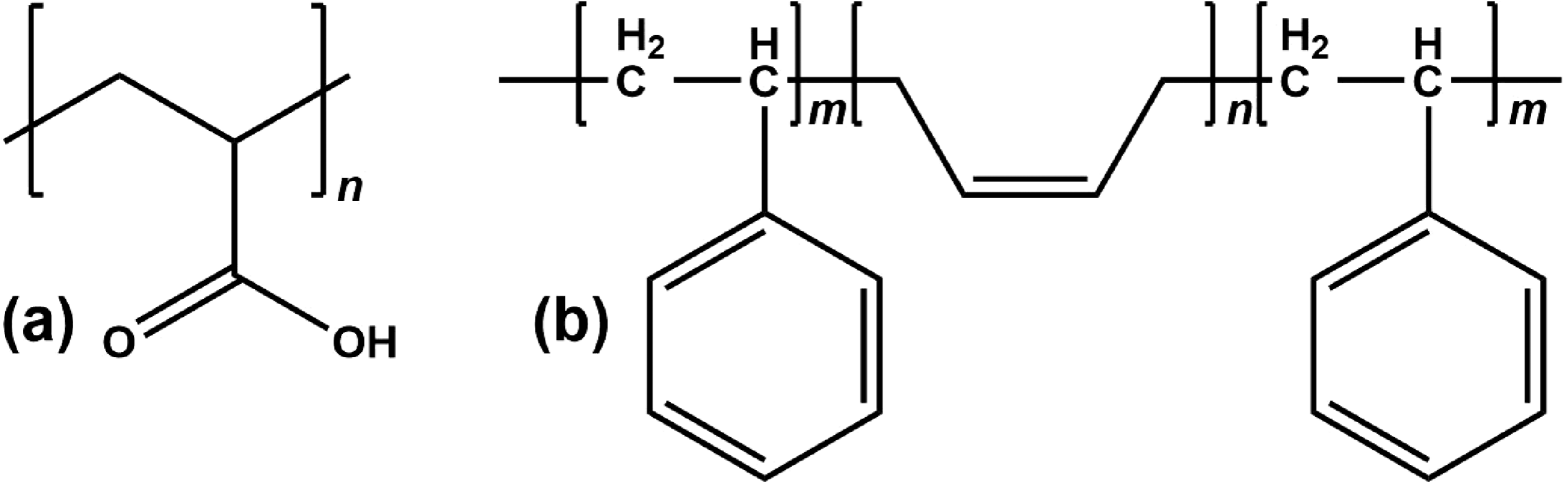
Chemical structures of (a) poly(acrylic acid) (PAA) and (b) polystyrene-*block*-poly-*cis*-butadiene-*block*-polystyrene (SBS).

Poly(acrylic acid) (PAA;  450 kg/mol, Figure 1a), a polymer soluble in both water and ethanol, was purchased from Sigma-Aldrich and either dissolved in anhydrous ethanol (purchased from VWR) to prepare a 10% w/w solution or in ultrapure deionized water (Sartorius Arium system, resistivity 18.2 MΩ·cm) to make an 11.5% w/w PAA–water solution. Polystyrene-*block*-poly-*cis*-butadiene-*block*-polystyrene (SBS; 30% w/w styrene;  140 kg/mol, Figure 1b) was also purchased from Sigma-Aldrich and dissolved in tetrahydrofuran (THF, from Sigma-Aldrich) to prepare a 10% w/w solution. For the core material, we used the multicomponent nematic liquid crystal mixture RO-TN 651 (proprietary composition), sourced from F. Hoffman-La Roche (Basel, Switzerland), on its own or mixed with 10% w/w of ethanol. We measured the surface tension of pure RO-TN 651 to be 32.33 ± 0.02 mN/m (at 20 °C); the surface tension of ethanol (at 20 °C) is 22.31 mN/m.^68^ We could not measure the surface tension of the mixture of RO-TN 651 with 10% w/w ethanol because this mixture wets the needle used to make a pendant drop (even when a needle made from poly(tetrafluoroethylene), PTFE is employed). Nevertheless, we expect that the surface tension of the ethanol/RO-TN 651 mixture falls within the range of 22–32 mN/m, bounded by the values corresponding to the pure components. All materials were used as received without further purification.

### Electrospinning Parameters

The dual-phase spinneret used for electrospinning, consisting of coaxially mounted stainless steel needles (external/internal diameter of the inner needle: 0.9/0.6 mm; of the outer needle: 1.7/1.4 mm), was purchased from Y-Flow. The outer needle of the spinneret has dents to keep the inner needle in the center and, at the same time, to provide an ohmic contact between the two needles that ensures they are at the same electrical potential. The spinneret was stored in ethanol when not in use and, prior to and after experiments, was thoroughly rinsed with fresh 96% w/w ethanol to remove any material residues. Before starting the electrospinning process, the spinneret was carefully dried to avoid any possible cross contamination from the lower-grade ethanol used for cleaning. This was achieved by flushing the spinneret with compressed air and storing it at 25 °C for a few hours.

**Figure 2:**
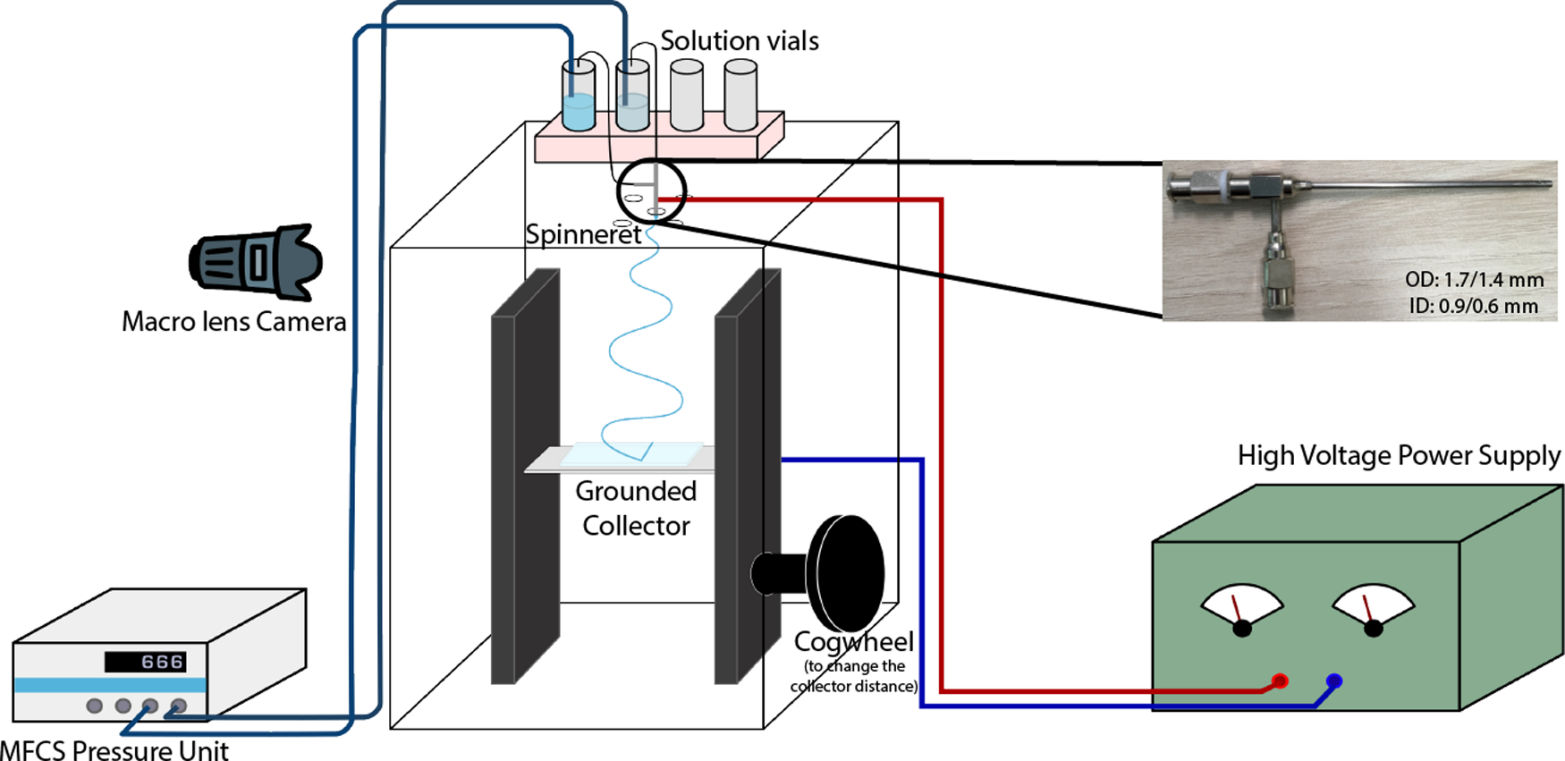
Schematic representation of the electrospinning setup. MFCS is the pressure control unit that controls the liquid flow. Inset: The spinneret used for the experiments.

**Figure 3:**
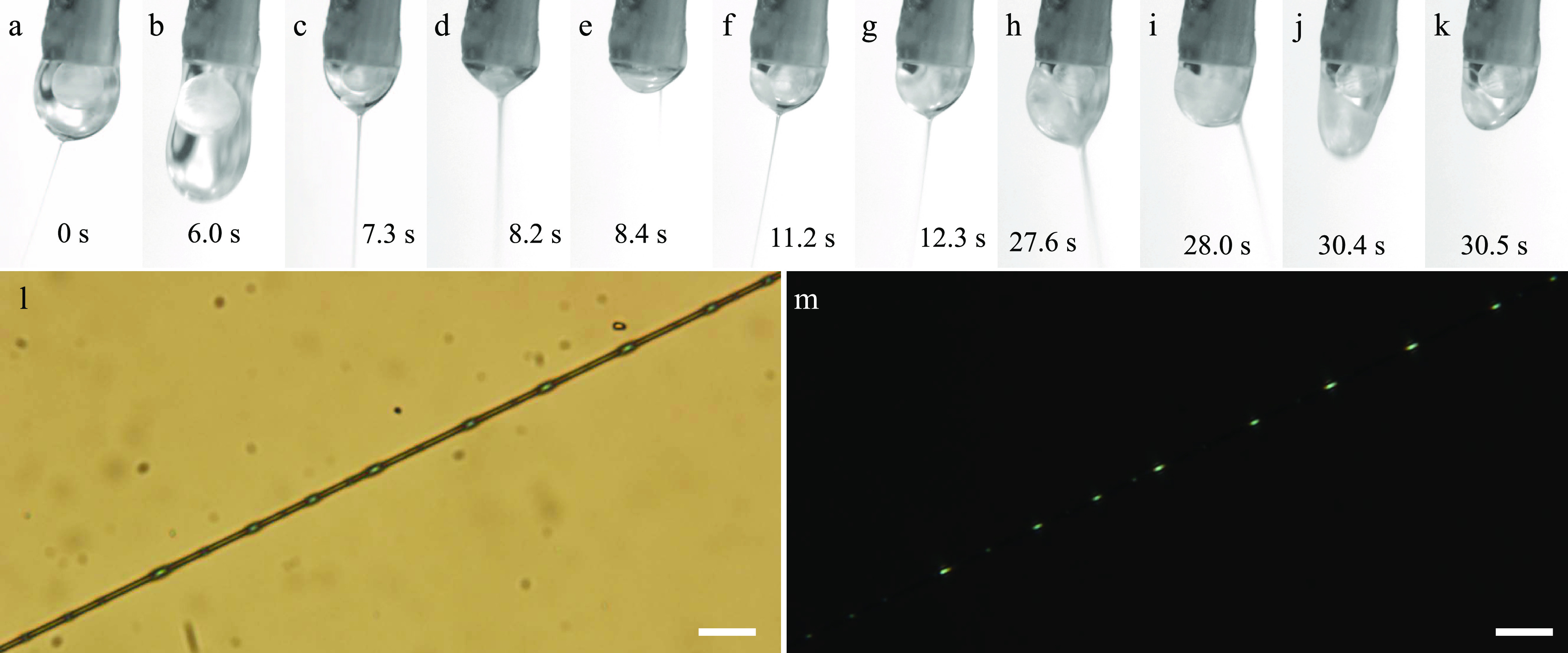
Top row: Taylor cone at different stages during electrospinning the LC core into an 11.5% w/w PAA-in-pure water sheath solution. The images are extracted from Supporting Information Movie S1, the time stamps relating to the start of the movie. The outer needle of the spinneret (1.7 mm diameter) appears slightly inclined at the top of every image because the camera was not oriented perfectly; in reality, the spinneret was vertically oriented. Bottom row: polarizing optical microscope (POM) images (l: without analyzer; m: between crossed polarizers) of the best-quality fiber produced during this experiment, exhibiting regularly spaced beads filled with LC. Scale bars are 20 μm.

Figure 2 shows a schematic representation of the electrospinning setup. It is housed inside an acrylic box, with a mobile collector wrapped in aluminum foil and the spinneret inserted with vertical needle orientation from the top. The fluids are pumped to the respective spinneret needle through tubes connected to fluid vials pressurized by a microfluidic pressure unit (Fluigent, model MFCS-EZ, maximum pressure 1034 mbar, uncertainty ± 0.3 mbar), and the electrical potential of the spinneret is controlled by connecting the outer needle to a high-voltage power supply (γ High Voltage, model ES30R-5W/DAM/RS232). The Taylor cone was imaged using a macro lens (Tokina AT-X Pro) mounted on a camera (Pixelink D755). Representative still frames were extracted from the movies and then digitally enhanced for clarity using “Adjust image” in Keynote (Apple), setting Saturation at −100%, the right-most Levels parameter to 53%, and the middle one to 40% (Figures 3, 5, and 6) or 47% (Figure 7). In Figures 3, 5, and 6, Exposure and Shadows were additionally adjusted to 100%.

Fibers were collected freely hanging on a copper wire frame and on hydrophobized glass microscopy slides to avoid wetting and collapse of the filled fibers.^27,69^ These slides were prepared by cleaning 25 mm × 75 mm borosilicate glass microscopy slides (Carl Roth) with alternating rinses of isopropanol and ultrapure deionized water before surface activation with a handheld corona generator for at least 30 s. The plasma-treated slides were then immediately immersed in an aqueous solution of 2% v/v *N*,*N*-dimethyl-[*N*-octadecyl-3-aminopropyl]trimethoxysilyl chloride (DMOAP, 42% in methanol, Sigma-Aldrich) and allowed to stand for at least 15 min, with gentle shaking halfway through the soaking procedure to ensure that the solution adequately coated and functionalized the glass slides. The slides were then removed from the treatment solution, rinsed several times with deionized water, and dried under vacuum at 120 °C for at least 30 min.

### Establishment of Phase Diagram between RO-TN 651 and Ethanol

**Figure 4:**
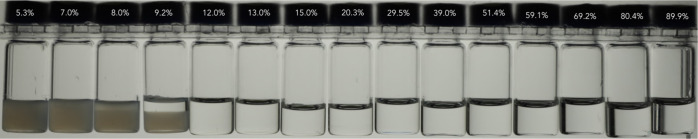
Sequence of vials with RO-TN 651 and gradually increasing concentration of anhydrous ethanol from left to right; the indicated percentages at the top refer to the mass fraction of ethanol. Because the nematic phase is turbid and sinks to the bottom, it is easy to recognize, although the phase separation had not completed in the 8.0% sample at the time of photography (about 20 min after the last sample preparation). We conclude the existence of a miscibility gap extending from around ∼7.5% w/w to around ∼11% w/w ethanol.

Vials of volume 2 mL were half-filled with each mixture, prepared by measuring out target volumes of first LC and then ethanol using Eppendorf pipettes and weighing the sample after each addition step. To minimize evaporation of ethanol and condensation of water, the vials were closed immediately after addition of the ethanol. After all samples had been prepared, each vial was shaken vigorously for about 1 min on a vortex mixer to ensure complete mixing. After this, the series of sealed vials were left to stand overnight in an air-conditioned lab with the temperature set to 21 °C, before the photograph shown in Figure 4 was captured.

### Optical Characterization

Once collected, the fibers were optically characterized using a polarizing optical microscope (POM; Olympus BX-51) with a camera (Olympus DP73). POM characterization was carried out in transmission mode between crossed polarizers or with the analyzer removed.

### Interfacial Tensiometry

Interfacial tension measurements were performed using a pendant drop tensiometer (Goniometer OCA 15EC from Dataphysics). The density of the solutions for interfacial tensiometry was measured using a Mettler Toledo DE45 Delta range densitometer. Both density and interfacial tension measurements were performed at room temperature.

## Results and Discussion

### Coaxial Spinning with Aqueous PAA Sheath and LC Core: Impact of Excessive Interfacial Tension

When attempting to electrospin the LC RO-TN 651, which has negligible miscibility with water, as a core inside the PAA–water sheath solution, the relatively high γ_cs_ (measured to be 9.13 ± 0.3 mN/m at 20 °C; see Supporting Information Movie S5) causes significant problems, as seen from a detailed frame-by-frame analysis of Supporting Information Movie S1, showing the Taylor cone dynamics during a run with flow rates optimized for maximum fiber filling. Representative still frames are shown in Figure 3a–k. It is still possible to spin fibers with encapsulated LC in this way (Figure 3l–m), but we cannot get a continuous LC core and it is a very lossy process since the required overfilling of the Taylor cone with LC (to be explained below) means that the majority of the LC pumped to the spinneret never makes it into the jet.

We initially pump only the sheath solution, starting the injection of the LC core solution once a stable PAA–water Taylor cone with consistent spinning has been established. As seen at the beginning of Supporting Information Movie S1 and in Figure 3a, the LC pumped from the inner needle forms a nearly spherical droplet inside the external sheath solution, hovering far above the Taylor cone apex from which the jet is ejected. The fibers produced at this stage of the process are thus devoid of LC since no LC makes it into the jet. Because we use a high LC flow rate, the inner droplet and the overall Taylor cone continuously grow in volume and, about 2 s into the movie, the jet stops: most likely, there is too large a voltage drop from the spinneret to the bottom of the droplet at this size. Now both sheath and core droplets grow until ∼6 s into the movie, when the sheath droplet rapidly elongates before it is cleaved (Figure 3b). The cleavage process detaches the entire LC droplet from the spinneret, and most of it—but not all—leaves the Taylor cone together with the detached sheath solution.

The remainder of the LC separated from the spinneret forms a small droplet near the bottom of the Taylor cone, from which a stable jet is again ejected. The suction from the jet pulls the bottom LC droplet toward it until that in Figure 3c connects to the Taylor cone apex such that the jet is now injected with LC. In the meantime, a new top droplet of freshly injected LC has started growing from the spinneret. Once this has become large enough to touch and merge with the bottom LC droplet, we have a brief moment with a single LC volume that extends continuously from the inner spinneret needle all the way to the jet, thus yielding an ideal coaxial Taylor cone (see Figure 3d). However, this shape of the LC volume does not minimize the core–sheath interface area, and therefore the interfacial tension (γ_cs_ = 9.13 ± 0.3 mN/m; see the Supporting Information) renders this an unstable equilibrium. The continuous LC flow very quickly collapses into a geometry with a bottom LC drop again detached from the spinneret needle; see Figure 3e. At the same time, the jet moves from the bottom of the Taylor cone to the boundary between LC and sheath solution.

The lower LC droplet—which is now larger than before due to the merger with the new LC—moves to the left, detaching from the jet, which thus again contains no LC (Figure 3f). A new droplet grows from the inner spinneret needle until it merges with the lower LC droplet (Figure 3i), which thereby acquires a size large enough that it extends past the cone apex, hence now LC is again fed into the jet. This cycle of new LC droplet growing from the spinneret needle until it is large enough to merge with the LC droplet residing at the bottom of the Taylor cone, thereby separating from the spinneret and leaving an increasingly larger lower LC droplet, repeats itself another five times, the last two steps shown in Figure 3h–i. Throughout this stage, the jet is continuously fed with LC as the Taylor cone apex is fully covered by the lower LC droplet. However, since the process requires overfeeding of the Taylor cone with LC, the process makes the inner LC droplet, and thus the overall Taylor cone, increasingly heavier, and in Figure 3j, another cleavage event occurs, removing most of the LC from the Taylor cone. Again, a fraction is left at the bottom of the Taylor cone (Figure 3k) and the full cycle repeats itself.

While this trick of overfeeding the Taylor cone with LC thus leads to core injection into the jet a large fraction of the time, it comes at the cost of very significant loss of LC every time a droplet is pinched off from the Taylor cone, in addition to the problems that the macroscopic drop may cause if it lands on the fiber mat. Note that horizontal electrospinning cannot be used in this mode, since we rely on gravity to push the detached lower LC droplet on top of the jet. Moreover, even during the period when the jet is fed with LC, the relatively high γ_cs_ continues to cause problems within the jet, triggering an internal Rayleigh–Plateau instability that breaks up the continuous core into a train of discrete LC droplets. The result is that the best fibers produced with this core–sheath combination are beaded, with discrete pockets of LC regularly spaced along the fibers; see the example in Figure 3l–m.

### Replacing Water with Ethanol in the Sheath Solution

While RO-TN 651 is practically insoluble in water, the mixture is partially soluble in ethanol. Figure 4 shows that about 7.5% w/w anhydrous ethanol destabilizes the nematic phase at room temperature, and the miscibility gap between the ethanol-poor nematic phase and ethanol-rich isotropic phase extends to between 9.2 and 12.0% w/w. Although we cannot establish the mole percentages since we do not know the composition of the commercial RO-TN 651 mixture, the miscibility gap appears to be somewhat narrower than that of ethanol and the commonly used single-component LC 5CB, which at room temperature extends from ∼13 to ∼23 mol %.^70^ At no point do we see two isotropic phases in coexistence in Figure 4. The partial miscibility of RO-TN 651 and ethanol, and the miscibility gap starting at low ethanol concentrations, render an experiment using PAA dissolved in ethanol as sheath and RO-TN 651 as core highly interesting. The miscibility gap ensures that a transient, yet well-defined interface exists between core and sheath, even if they start mixing, as the core is continuously replenished with fresh LC from the spinneret. At the same time, the nonzero miscibility means that the phases on both sides of the interface contain the same chemical substances, only at different concentrations. We can thus expect a much lower γ_cs_ than the ca. 9 mN/m measured for the case where water-dissolved PAA is the sheath solution. We indeed confirm this while attempting to measure the interfacial tension of a pendant RO-TN 651 drop in a bath of a PAA/ethanol solution. Although a stable RO-TN 651 drop cannot be formed at equilibrium, a drop with a well-defined fluid interface is formed while the LC phase is ejected from the needle into the polymer solution. When the LC flow is stopped, the LC drop slightly increases in size (presumably due to ethanol from the bath mixing with the LC) and the boundary between the two fluids becomes decreasingly sharp (see Figure S1, Movie S6, and the corresponding discussion).

**Figure 5:**
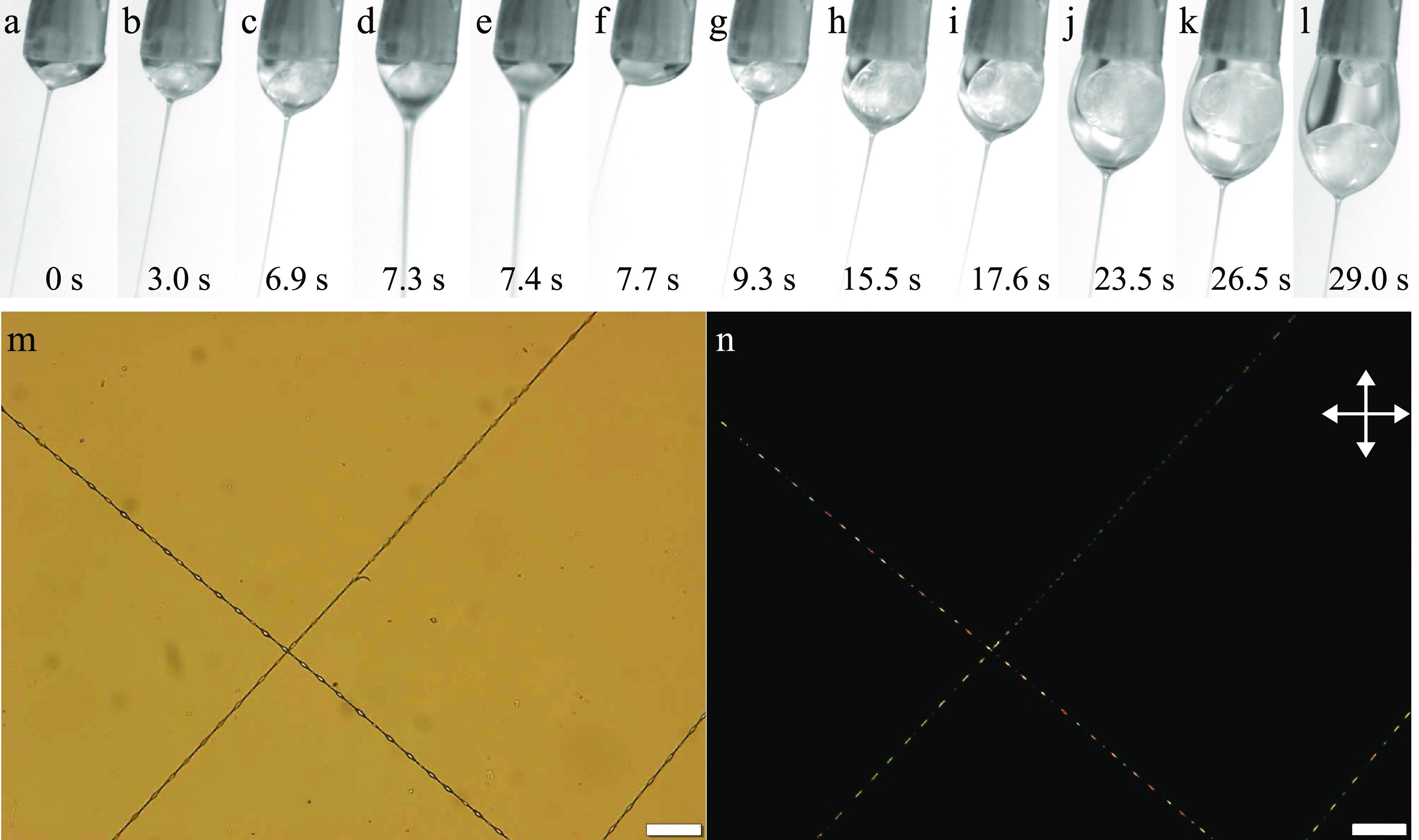
Top row: Taylor cone at different stages during electrospinning the LC core into a 10% w/w PAA in pure ethanol sheath solution. The images are extracted from Supporting Information Movie S2, the time stamps relating to the start of the movie. The outer needle of the spinneret (1.7 mm diameter) appears at the top of every image, slightly inclined because the camera was not oriented perfectly; in reality, the spinneret was vertically oriented. Bottom row: POM images (m: without analyzer; n: between crossed polarizers, as indicated by double-headed arrows) of the best-quality fiber produced during this experiment, exhibiting regularly spaced beads filled with LC. Scale bars are 50 μm.

The result can be seen in Supporting Information Movie S2, with representative still images collected in Figure 5, showing that the situation is still far from ideal. Initially (Figure 5a–c), the inner LC core can be distinguished, as being surrounded by an increasingly turbid mixed phase that grows with time from bottom to top of the Taylor cone. During this stage, the core LC appears to be disconnected from the jet, leading to its continuous increase in volume until it connects to the jet in Figure 5d. The jet suddenly broadens greatly as much of the collected LC is ejected from the Taylor cone which rapidly diminishes in size (d–f). Around 10 s into the movie (Figure 5g–h), flow patterns are clearly seen, and the LC droplet appears to disconnect from the jet. The LC is still attached to the spinneret, however, so the droplet hovers further and further above the apex of the Taylor cone as the latter continues to grow in size (i, j). The LC eventually detaches from the spinneret 26.5 s into Supporting Information Movie S2 (Figure 5k), settling at the bottom of the Taylor cone in Figure 5l, after which the jet is again fed with LC. The situation now resembles that of Figure 3, with a bottom LC droplet resting on top of the jet ejection point and a top droplet attached to the spinneret needle growing in size until the two LC volumes merge and/or the Taylor cone becomes so large that it detaches from the spinneret.

We believe that the problems seen in this experiment arise because γ_cs_ is not constant within the Taylor cone: we expect a continuous gradient in γ_cs_ from the top of the spinneret, where pure LC comes in contact with pure PAA/ethanol solution, to the point further down along the core–sheath interface where enough mixing of the two liquids should have occurred to reach the miscibility gap. From this level and downward, we can expect an extremely low γ_cs_ between the coexisting nematic and isotropic phases. As a result of this spatial variation in γ_cs_ along the axis defined by the spinneret, we anticipate that the solutal Marangoni effect^71,72^ sets up a new internal flow along the internal LC–polymer solution interface. Since this flow is directed from low to high γ_cs_, it is counter-directed to the top–down flow from the spinneret (assuming that the LC droplet is in contact with both the spinneret and the apex of the Taylor cone). We thus get a circular flow pattern around the core–sheath interface, with the innermost LC moving downward while the interface is moving upward, promoting mixing and disturbing the interface, in turn causing new Marangoni stresses. This circular flow can be visualized in Supporting Information Movie S2.

In parallel, we expect to have another Marangoni effect-driven flow at the outer surface of the Taylor cone (i.e., at the air–PAA/ethanol interface). Cooling due to evaporation of ethanol leads to water condensation from the atmosphere,^67^ rendering the surface tension between sheath solution and air, γ_sa_, higher at the bottom of the air–PAA solution interface than at the top, where fresh ethanol solution without water emerges from the spinneret. This flow is from the top to the bottom (along the outer interface), thus reinforcing the natural flow within the sheath solution. In summary, we thus have the pressure-induced downward-directed flow at the very center of the Taylor cone where fresh LC is injected as core liquid; upward-directed flow at the core–sheath interface thanks to the solutal Marangoni effect; and downward-directed flow at increased speed along the Taylor cone outside, given by the sum of the pumped-out sheath solution and the thermal Marangoni effect. We can thus expect a highly complex process with multiple vortices within the Taylor cone, in combination with mixing of water into the sheath solution from the outside and a certain degree of mixing with core at the inside. Elaborate flow visualization experiments are needed to draw clear conclusions about the features of both the solutal and thermal Marangoni flows (e.g., their strength) and their interplay with the other hydrodynamic patterns inherently involved in the electrospinning process; while such experiments would be highly interesting, they are beyond the scope of this study.

The produced fibers are again beaded, with LC within the beads; see Figure 5m,n. This suggests that the mixing of ethanol from the sheath into the LC core is not fast enough to significantly reduce γ_cs_ below the level where it triggers the Rayleigh–Plateau instability within the jet prior to sheath solidification. Indeed, when preparing the experiment in Figure 4, we noticed that diffusion of LC and ethanol across the nematic–isotropic phase boundary is slow, motivating the active vortex mixing. Even if the Marangoni effects induce some active mixing in the Taylor cone, this is not enough to give the core and sheath fluids forming the compound jet so much of each other’s constituents that γ_cs_ loses its impact. To reach that state, we need to adjust the core composition already prior to spinning.

### Optimum Spinning Conditions by Adding Ethanol also to the Core

**Figure 6:**
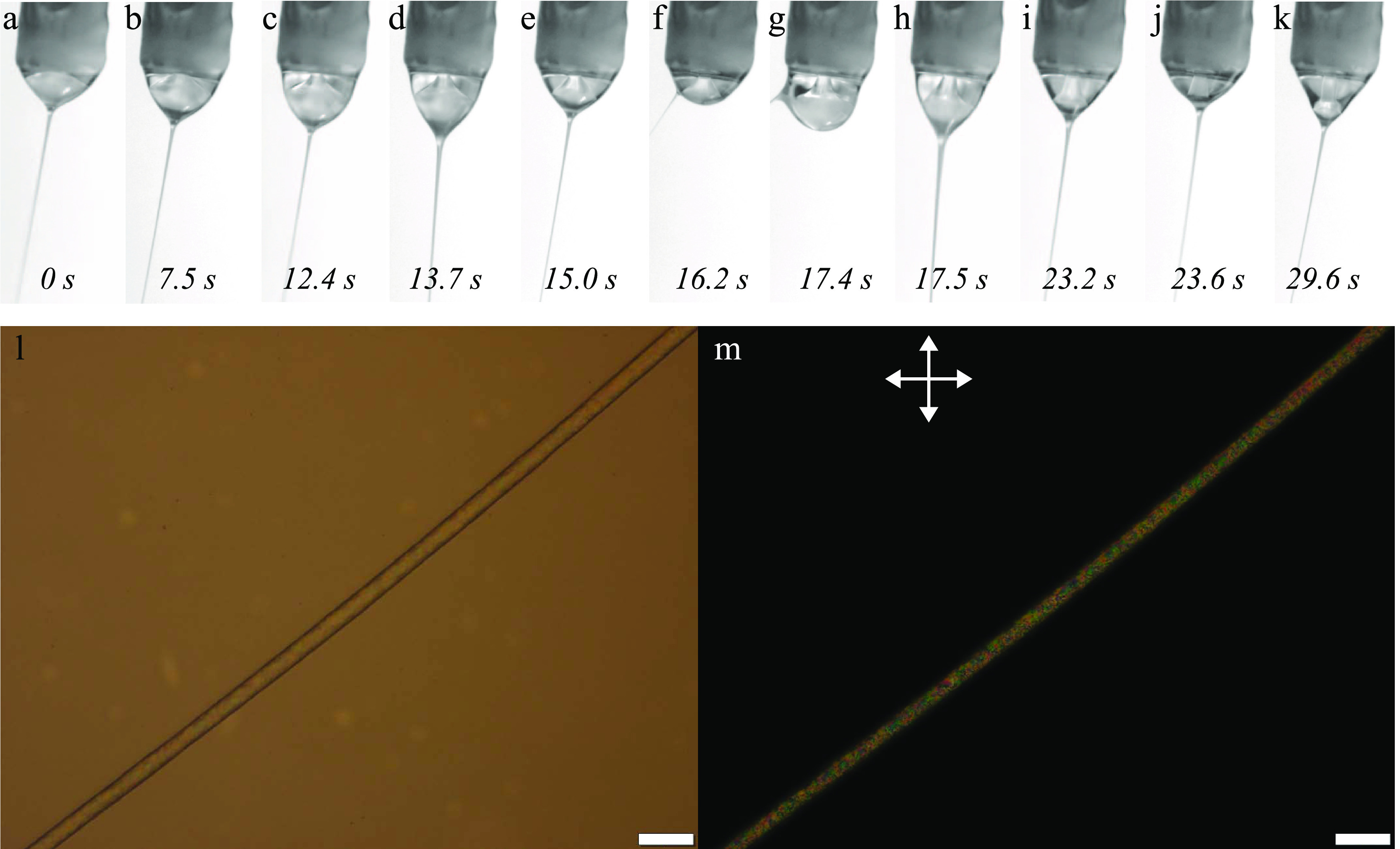
Top row: Taylor cone at different stages during electrospinning a core of LC with 10% w/w of ethanol added into a 10% w/w PAA in pure ethanol sheath solution. The images are extracted from Supporting Information Movie S3, the time stamps relating to the start of the movie. The outer needle of the spinneret (1.7 mm diameter) appears at the top of every image, slightly inclined because the camera was not oriented perfectly; in reality, the spinneret was vertically oriented. Bottom row: POM images (m: without analyzer; n: between crossed polarizers, as indicated by double-headed arrows) of the best-quality fiber produced during this experiment, exhibiting continuous filling of LC. Scale bars are 50 μm.

To reduce the impact of Marangoni flow, we add 10% w/w of ethanol to RO-TN 651 and fill this into the vial for core liquid. Note that this has brought us more than halfway into the miscibility gap; hence, we can expect a minimum γ_cs_ between the nematic phase and its coexisting isotropic phase. It also means that we have phase separation in the reservoir from which the core liquid is pumped, but we know that we are pumping only the nematic phase as core because we use pneumatic pumping rather than syringe pumps, and we place the tip of the needle taking the core liquid at the bottom of the vial. Based on the experiment in Figure 4, we can estimate the amount of ethanol in the nematic phase in the miscibility gap, which is our core liquid in this new experiment, to be about 7.5% w/w. The advantage of this approach is that γ_cs_ is significantly reduced already when the two liquids first come into contact, a slight further reduction happening on the way down from the spinneret orifice as LC diffuses out into the ethanol-PAA solution, bringing its composition closer to that of the equilibrium isotropic phase bounding the miscibility gap. A gradient in γ_cs_, inducing Marangoni flow, thus still exists, as visible in Supporting Information Movie S3 (showing the full experiment) and in Figure 6 (summarizing the key observations), but it is not strong enough that the induced flow can disrupt the coaxial spinning. The effect could probably be canceled out completely by adding LC to the sheath solution until it has the composition of the isotropic phase in the miscibility gap, but as the LC is not a good solvent for PAA, this would cause other problems. We find that tuning the core composition to that of the miscibility gap boundary, while keeping the sheath solution free of LC, is sufficient to produce good coaxial fibers.

From an applied point of view, the single most important observation in Supporting Information Movie S3 is that the LC flow is uninterrupted throughout the entire experiment: we can easily confirm a continuous stream of LC from the inner spinneret needle orifice to the Taylor cone apex, and from there into the jet, in every frame. This is despite the fact that we exchanged the collector about halfway into the movie, causing significant temporary alterations of the electric field profile with strong shape changes of the overall Taylor cone shape. However, the experiment actually contains much more information, revealing important data on the phase separation and the interfaces present within the Taylor cone, as the following detailed analysis highlights.

When the core enters the Taylor cone, it quickly builds up a turbid volume from the bottom of the Taylor cone, filling most of it in Figure 6a. Over the next few seconds, the overall Taylor cone grows vertically downward, the lower turbid LC volume retaining a roughly constant size and moving downward, with a narrower stream of core flow connecting it to the inner spinneret needle; see Figure 6b–d. At the same time, it becomes increasingly clear that a near-horizontal boundary between two isotropic phases exists, separating the nearly pure sheath solution freshly emerged from the outer spinneret needle from the lower Taylor cone part, which has an air interface with weaker curvature just below the boundary, best seen in panel (b). We thus have two phase boundaries in the Taylor cone: a lower one between nematic and isotropic phases, rich and poor in RO-TN 651, respectively, and an upper boundary between two phases that are both isotropic. The latter type of phase separation is not present in the mixtures of RO-TN 651 and pure anhydrous ethanol, as seen in Figure 4. We conjecture that the presence of PAA in the sheath solution creates a small second miscibility gap, between the pure PAA-in-ethanol sheath solution emerging from the spinneret and the slightly LC-enriched isotropic ethanol-PAA solution that is in equilibrium with the nematic core. Another possibility is that water condensing onto the Taylor cone from the air^67^ shifts the phase diagram to such an extent that an isotropic–isotropic phase separation takes place at very low LC concentration, similar to what is seen with 5CB–ethanol solutions.^70^ Importantly, with RO-TN 651 as core, neither phase boundary destabilizes the spinning process, so the interfacial tension of each boundary must be very low.

The LC flow is generally clearer at the top than at the bottom, probably because of shear alignment of the director as the LC exits the spinneret, but this alignment is lost when the core flow hits the boundary to air near the Taylor cone apex, leading to strong light scattering. The boundary between the isotropic phases moves upward and is almost flush with the spinneret in panels (c)–(d), but, in panel (e), it has moved down a bit. Around this time in the experiment, we switched to a different collector, temporarily yet considerably distorting the electric field. As a result, the Taylor cone gets smaller and, in panel (f), the symmetry is broken, the jet moving to one side. The Taylor cone distortion reaches its extreme situation in panel (g). In panel (h), the new collector is in place and the jet moves down to the bottom of the Taylor cone, which is now nearly cylindrically symmetric. Over the next few seconds, the Taylor cone shrinks somewhat again, adopting a true cone shape in panel (i), where the horizontal upper phase boundary is easily distinguished. The Taylor cone fluctuates slightly in size after the collector switch, reaching its minimum size in panel (j). In panel (k), the final steady-state situation is shown, with a Taylor cone that is largely conical in shape, a distinct horizontal isotropic–isotropic boundary just below the spinneret orifice, and an LC core flow that is almost cylindrical throughout the top two-thirds of the Taylor cone, broadening only near the bottom.

Throughout the whole process, the core flow is uninterrupted, and the jet only experiences the moving-around at the height of the disturbance due to the collector change. Apart from this moment (lasting about a second), a continuous core–sheath jet is ejected from the apex of the Taylor cone. Also the produced fibers are continuously—and richly—filled with LC (Figure 6l–m), demonstrating that γ_cs_ is too low to trigger the Rayleigh–Plateau instability within the jet. Our attempts to measure γ_cs_ of this system using a pendant ethanol/RO-TN 651 drop in a PAA/ethanol bath failed: a stable drop could not be formed (see Supporting Information Movie S8 and the discussion in the Supporting Information). Nevertheless, the very low effective interfacial tension is clear from the nonminimizing behavior of the interface. Since any remaining ethanol in the core is easily evaporated after spinning, its presence during the spinning process will not affect the behavior of the LC core when the fibers are used.

### Case of Complete Core–Sheath Miscibility

If one only considers the impact of γ_cs_, the best option might appear to be to eliminate the interface entirely by choosing a core and a sheath that are fully miscible, as then a smooth concentration gradient can form all the way from pure sheath to pure core without any discontinuity. Without an interface, there is also no interfacial tension and the problems encountered so far will not arise. However, this option leads to other problems: first of all, the loss of core–sheath structure that already Li and Xia noted in their pioneering work.^16^ This is particularly critical when—as in the present work—the core is a low molar mass liquid, since then we cannot rely on the low miscibility of polymeric solutes to prevent or at least slow down core–sheath mixing. Even more critically, if core and sheath are fully miscible but the core is not a good solvent for the sheath polymer (typically the case with functional core materials like LCs), then the loss of sheath solvent into the core and core liquid moving into the sheath will rapidly deteriorate the quality of the sheath solution from the perspective of dissolving the polymer. The core effectively becomes an internal coagulation bath, as used in traditional wet spinning to rapidly solidify the polymer. If the sheath solvent is volatile, the polymer concentration in the sheath solution also rapidly decreases and/or water is condensed from the air, adding yet another nonsolvent for many polymers. The combined result is that the Taylor cone is strongly distorted and often clogged within seconds or minutes, disrupting the spinning process.

**Figure 7:**
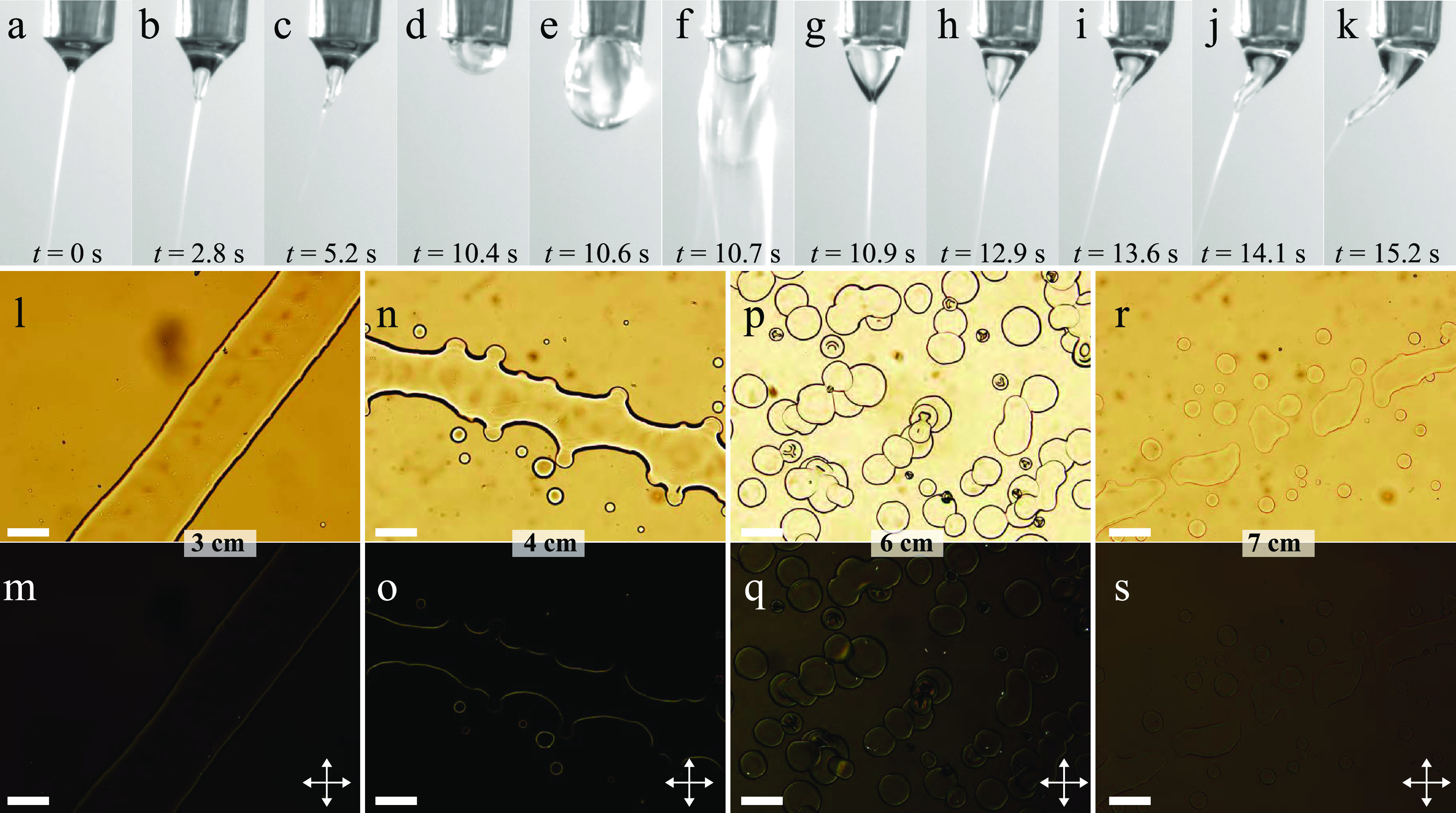
Top row: Taylor cone at different stages during electrospinning a core of RO-TN 651, with 10% w/w added THF, into a 10% w/w SBS in THF sheath solution. The images are extracted from Supporting Information Movie S4, the time stamps relating to the start of the movie. The outer needle of the spinneret (1.7 mm diameter) appears at the top of every image, slightly inclined because the camera was not oriented perfectly; in reality, the spinneret was vertically oriented. Bottom rows: POM images (l, n, p, r: without analyzer; m, o, q, s: between crossed polarizers, as indicated by double-headed arrows) of the spinning product collected at 3, 4, 6, and 7 cm below the spinneret, respectively. Scale bars are 10 μm.

To demonstrate these problems, we choose styrene-butadiene-styrene (SBS) block copolymer dissolved in tetrahydrofuran (THF) as a relevant example of a sheath solution that is highly miscible with RO-TN 651. This sheath is interesting because several groups have successfully electrospun SBS dissolved in THF (and co-solvents) into highly stretchable elastomeric fiber mats,^73^ which may then serve as a basis for stretchable electronic composites,^74^ light-emitting diodes (LEDs),^75^ or wearable organic vapor sensors.^76^ We have ourselves tried to use SBS as a sheath for LC core-functionalized fibers (with and without addition of dimethylformamide (DMF) as a co-solvent), but without success. In the experiment shown in Supporting Information Movie S4 and Figure 7, the reasons for the failure are clearly revealed.

During the first 10 s of the experiment, only sheath solution is spun and we initially see an excellent Taylor cone in Figure 7a. However, even without any core being injected, we see that THF as the sole solvent is not ideal: some 2 s into the movie, the tip of the Taylor cone starts elongating (b), clearly demonstrating that it is no longer in a fully liquid state. The volatile THF evaporates too quickly, and probably the consequent cooling of the Taylor cone also induces condensation of water,^67^ which dissolves in THF but is a nonsolvent for SBS. The result is gelation of the Taylor cone toward its apex, which, in panel (c), even gives it a clearly asymmetric distortion.

Because of the gelation, the spinneret is wiped clean (seen in Supporting Information Movie S4) and directly afterward (d) we start the injection of the LC core, easily recognized inside the Taylor cone by its turbid character. This initially stops the ejection of the jet and the compound Taylor cone grows in size (e) until the sheath breaks, without breaking off the core (f). Directly afterward, at 10.9 s into the movie (Figure 7g), a good core–sheath Taylor cone can be seen, with the core extending continuously from the spinneret orifice to the Taylor cone apex, from which a compound jet is ejected. However, already in panel (g) one can notice a horizontal boundary of the sheath just below the spinneret orifice, and in panel (h), we clearly see that the Taylor cone curvature is different above and below this boundary. This suggests that the sheath solution starts to gel below the boundary, allowing the Taylor cone to extend more vertically here than above the boundary. Importantly, this boundary was never seen prior to injecting the LC (see panels a–c); hence, it is the core acting as coagulation bath that is causing this deterioration of the Taylor cone.

In panel (h), we can even start distinguishing a second boundary further down, which becomes very clear in panel (i). Below the second boundary, the Taylor cone loses its cylindrical symmetry and it distorts leftward in the image. The distortion increases in size over the next few seconds (j) until that in panel (k) is so strong that reliable spinning from the Taylor cone is no longer possible. We wipe the spinneret clean just after (k), but it takes less than 4 s until the Taylor cone again deforms so strongly that spinning is stopped; see Supporting Information Movie S4. Although the rapid evaporation of the volatile THF already causes problems for the single-phase spinning (this is probably why Fong and Reneker^73^ and Park et al.^74^ used THF–DMF solvent mixtures for spinning), we see that the situation is dramatically worsened by the introduction of the core liquid that is fully miscible, without any miscibility gap, with the sheath solvent.

The elimination of core–sheath interface in the Taylor cone indeed has the advantage that the core is drawn very well into the jet, even when the Taylor cone is as distorted as in Figure 7k. But this is of little use to us: first, because it is only a matter of seconds until spinning is stopped due to Taylor cone gelation, and, second, because the core–sheath structure is lost in the jet. This is seen in panels (l)–(s), showing samples that have been collected on glass slides at different distances below the spinneret. A slide held at only 3 cm below the spinneret shows continuous ribbons, but they are very broad as no significant jet stretching could take place and much liquid still remains in the fiber; see Figure 7l–m. Importantly, there is no trace of a core–sheath structure, and the image between crossed polarizers (m) reveals that there is no liquid crystalline behavior at any point in the ribbon.

Moving further down to 4 cm below the spinneret (n–o), the ribbon has strongly undulated edges, indicating that the Rayleigh–Plateau instability is about to break it into droplets. Indeed, several droplets surround the broad ribbon that runs across the image from left to right. As before, there is neither any sign of core–sheath structure nor of liquid crystalline behavior. At a 6 cm distance (p–q), the instability has entirely broken up the jet and we see only large droplets, and at 7 cm (r–s), we see some smaller droplets and some larger regions where nearby droplets have apparently merged with—as usual—no sign of core–sheath structure or of liquid crystalline behavior. Note that much of the volatile THF should have evaporated at this point, and, in experiments without an LC core, we have indeed frequently succeeded in spinning SBS fibers in this way. However, with an LC core, its complete miscibility with the sheath solution and its non-solid state mean that the sample remains liquid, removing any trace of fibers at this stage. While the LC is not a good solvent for SBS, it is compatible enough to act as a plasticizer.

### Comparison with Earlier Studies

We end by briefly revisiting some of the previously published papers discussed in the beginning in light of the new knowledge brought about by experiments. Considering first the early papers with miscible solvents by Sun et al.^14^ and Yu et al.,^15^ we note that both deal with polymer solutions as cores, with the exception of one experiment by Sun et al. where the core was a THF solution of palladium(II) acetate. The entangled nature of polymer solutions and the general difficulty to mix two different polymers makes it credible that no significant mixing between core and sheath polymers takes place during the residence time in the Taylor cone when core and sheath are both polymeric. We also note that the solvents of the core and sheath solutions can be grouped into three categories: (i) identical solvents or solvent mixtures (all cases in ref (15) belong to this category), (ii) immiscible solvents (e.g., water and chloroform) but with the addition of a co-solvent that is miscible with both other solvents (in the same example ethanol in water), and (iii) different miscible solvents, but neither is a nonsolvent for any polymer (e.g., a PLA-dichloromethane sheath solution was used with the palladium acetate-THF core solution in one of the experiments of Sun et al.,^14^ but PLA is soluble also in THF). Cases (i) and (iii) obviously give no relevant γ_cs_, whereas case (ii) has γ_cs_ greatly reduced by the presence of the co-solvent soluble in both phases.

We can thus conclude that these situations all avoid phase separation and Rayleigh–Plateau instabilities by keeping γ_cs_ very low; gelation is avoided by not using any nonsolvents for the polymers used; and loss of core–sheath structure is avoided by ensuring that the time from core and sheath meeting until the fiber sheath solidifies is kept much shorter than the characteristic mixing time of core and sheath solutions. Also the fabrication of hollow tubes and rods in tubes by Zussman et al.^61^ used polymeric core as well as sheath in identical solvents (DMF), but here also acetone was added as a co-solvent for the poly(methyl methacrylate) (PMMA) core solution. This is interesting since acetone is a nonsolvent for the PAN in the sheath; hence, the core acted as an internal coagulation bath, speeding up the solidification of the sheath. Obviously, some fine tuning is required to prevent this coagulation from starting prematurely in the Taylor cone; this may be why 40% DMF was included in the PMMA core solution.

In this context, a most interesting study is that by Luo and Edirisinghe of nonpolymeric core liquids, including water and glycerol, stabilizing electrospinning of polymer solutions as sheaths, when the same polymer solutions without a core only electrospray.^77^ They conducted a thorough study of miscibility of the components, finding that high γ_cs_ can lead to well-defined fibers. While they also pointed out initially that a high γ_cs_ promotes the Rayleigh–Plateau instability, they drew the conclusion that the fiber formation was supported by the high γ_cs_, although the mechanism for this was not clear. In light of our results and those of Zussman et al.,^61^ we believe that the main reason for the transition from electrospray to electrospinning when using water as core liquid is that it acted as an internal coagulation bath, since the sheath solvent was miscible with water. This was not the case when using glycerol as core, but here the much greater viscosity, 3 orders of magnitude greater than that of water, is likely to be important.

The encapsulation of an industrial oil (Elf SAE-15W50) as a core inside DMF-dissolved PVP by Díaz et al.^63^ and Díaz Gómez et al.,^62^ as well as inside water-dissolved PEO by Díaz Gómez et al.,^62^ is interesting, as Díaz et al. point out that low γ_cs_ is required,^63^ yet the oil–water interface when using aqueous PEO as sheath should have quite significant γ_cs_. However, the industrial oil actually contains surfactants;^63^ hence, this is an example where surfactant addition is a viable means of reducing γ_cs_. This approach is unfortunately not straightforward to use when working with LC cores because surfactants can strongly impact the LC alignment and even bring in emulsified water, with strong impact on the LC phase behavior.^27^ Despite the low γ_cs_ (values on the order of 1 mN/m were mentioned), the produced fibers were strongly beaded, but this may also be due to a mismatch in elongational viscosities between core and sheath.

The approach to use identical solvents in sheath and core to minimize γ_cs_ was used also by He et al.^44^ (PLA in hexafluoroisopropanol, HFIP, as sheath and the drug TCH in HFIP as core), but here the nonpolymeric nature of TCH could be expected to make retained core–sheath structure more difficult. The authors used a very high electric field (4 kV/cm), kept the core flow rate low, and let the inner needle protrude beyond the end of the outer needle in the coaxial spinneret. These are all features that minimize the residence time of core and sheath in the Taylor cone, which probably was kept small by the high electric field (no information about Taylor cone was provided). While López-Rubio et al. also used the same core and sheath solvent—water—in their study of bacterial inclusions in PVA sheath fibers, this fact was not discussed explicitly.^58^ However, since they obtained similar results using single-phase as with coaxial electrospinning, it is not obvious that a distinct core–sheath structure prevailed. In both cases, beaded fibers with bacteria contained in the beads were observed, but, given the size of the bacteria, this structure may have been driven by the bacterial cargo rather than by the coaxial spinning approach. Several studies with miscible or partially miscible cores and sheaths have made no detailed comments on the problems related to core–sheath stability,^30,34,37,41,43,45,46,49,53^ but we note that several fall into category (ii) or (iii) above, and it is not unlikely that the particular combinations were found empirically by trial and error.

Returning to the paper by Li and Xia,^16^ finally, which strongly promoted immiscible core and sheath, we note that the mineral oil used as core is actually not immiscible with the ethanol used as sheath solvent. The Food and Agriculture Organization of the United Nations states that mineral oil is “sparingly soluble in ethanol”,^78^ which means that this combination of core and sheath is ideal, as they are neither fully miscible nor immiscible, but have a miscibility gap, as with RO-TN 651 and ethanol studied by us here. Since the same holds for paraffin oil/wax, and also for chloroform and DMF as solvents, this explains the success of all other coaxial electrospinning papers stating a need for immiscible core and sheath,^17−19,38,42,66^ inspired by the original paper by Li and Xia. We can also conclude that these studies did, in fact, not work with immiscible, but with partially miscible liquids, but the significance of this distinction was not clear at the time. Other papers that emphasize the needs for immiscible core and sheath liquids, such as the microtube electrospinning by Dror et al.^65^ or the gene delivery fibers spun by Saraf et al.,^55^ achieve success by using mixed solvents, giving a common or at least miscible component between core and sheath.

## Conclusions

By comparing core–sheath electrospinning of a nonpolymeric and nonvolatile LC core in three different sheath solutions (one miscible, one immiscible, and one partially miscible with the LC), we have demonstrated that the optimum combination is partially miscible core and sheath liquids. When the two liquids have a miscibility gap in the phase diagram that does not reach all the way to either pure component, a distinct core–sheath interface can be maintained throughout the spinning process, yet the interfacial tension γ_cs_ can be kept very low, since both liquids contain the same chemical constituents, only at different compositions. This prevents phase separation in the Taylor cone, droplet formation due to a Rayleigh–Plateau instability in the jet, as well as loss of core–sheath structure due to complete mixing. In this way, the production of fibers with continuous core–sheath morphology can be ensured, of great value when the core adds functionality to the fiber. Importantly, the core should not be spun pure, but enough of the sheath solvent should be added to reach the miscibility gap of the phase diagram: otherwise, strong Marangoni stresses and, in turn, complex flow patterns can arise in the Taylor cone as a consequence of local variations of γ_cs_, preventing stable core–sheath spinning. While our experiments were conducted with an LC core, these conclusions are perfectly applicable to any other core–sheath combination.

## Supporting Information Available

Interfacial tension characterization of the spinning solutions; snapshots of the interfacial tension measurements using the pendant drop technique of RO-TN 651 to a solution of 10% w/w PAA in anhydrous ethanol (Figure S1); electrospinning parameters and conditions (Table S1) (PDF)
Taylor cone recorded during electrospinning RO-TN 651 as core and an aqueous solution of 11.5% w/w PAA as sheath (Movie S1) (MP4)
Taylor cone recorded during electrospinning RO-TN 651 as the core and a solution of 10% w/w PAA in anhydrous ethanol as sheath (Movie S2) (MP4)
Taylor cone recorded during electrospinning of 10% w/w anhydrous ethanol in RO-TN 651 core and a solution of 10% w/w PAA in anhydrous as sheath (Movie S3) (MP4)
Taylor cone during electrospinning of 10% w/w THF in RO-TN 651 as the core and a 10% w/w SBS in THF, as sheath solution (Movie S4) (MP4)
Interfacial tension measurement using the pendant drop technique with a drop of RO-TN 651 injected at 0.5μL/s into a bath of aqueous solution of 11.5% w/w PAA (Movie S5) (MP4)
Interfacial tension measurement using the pendant drop technique with a drop of RO-TN 651 injected at 0.1μL/s into a bath of 10% w/w PAA solution in anhydrous ethanol (Movie S6) (MP4)
Interfacial tension measurement using the pendant drop technique with a drop of RO-TN 651 injected at 0.05μL/s into a bath of 10% w/w PAA solution in anhydrous ethanol (Movie S7) (MP4)
Interfacial tension measurement using the pendant drop technique with a drop of 10% w/w anhydrous ethanol in RO-TN 651 injected at 0.1μL/s into a bath of 10% w/w PAA solution in anhydrous ethanol (Movie S8) (MP4)

## Footnotes

a Some refer to this as “triaxial” spinning, but this is unfortunately an incorrect and even misleading terminology; both the spinnerets and the fibers are uniaxial, as indeed required by a coaxial geometry.
