# Supplementary material for: Stable Electrospinning of Core-Functionalized Coaxial Fibers Enabled by the Minimum-Energy Interface Given by Partial Core–Sheath Miscibility
Source: Langmuir. 2021 Nov 4;37(45):13265–77. doi: 10.1021/acs.langmuir.1c01824 (PMC8600680; doi:10.1021/acs.langmuir.1c01824)
Supplement: Supplementary file 1 — la1c01824_si_001.pdf [file la1c01824_si_001.pdf]

Supplementary Information for "Stable electrospinning of core-functionalized coaxial fibers enabled by the minimum-energy interface given by partial core–sheath miscibility"

Shameek Vats[a], Manos Anyfantakis[a], Lawrence W. Honaker[a,c], Francesco Basoli[b], and Jan P.F. Lagerwall[a]

[a]*Experimental Soft Matter Physics Group, University of Luxembourg, L-1511 Luxembourg, Luxembourg*

[b]*Department of Engineering, Università Campus Bio-Medico di Roma, 00128 Rome, Italy*

[c]*Laboratory of Physical Chemistry and Soft Matter, Wageningen University & Research, 6703 DE Wageningen, The Netherlands*

Discussion about interfacial tension experiments

RO-TN 651-(PAA/water) system

To measure the interfacial tension of the RO-TN 651-(PAA/water) system, we used a stainless steel needle to make a pendant drop of the LC phase inside a bath of a PAA/water solution (11.5% w/w). The experiments were performed at 20∘C. From three independent measurements (i.e., using three different drops), we measured an average value of 9.13±0.30 mN/m (the error is the standard deviation).

The SI Movie 5 shows a representative example of such a drop. Even at very large volumes (up to 35 μL; beyond this volume the drop profile exceeds the imaging window), the drop is neither strongly deformed by gravity, nor can it be detached by the needle (unless we perturb it mechanically). This is attributed to the very low density difference between RO-TN 651 (1.0469 g/cm^3) and the PAA/water solution (1.0358 g/cm^3), respectively, which minimizes the effect of gravity on the drop. After we measured the interfacial tension in the stationary drop, we performed a few cycles of first pushing the needle against the drop, and then pulling it away from the drop. It can be clearly seen in SI Movie 5 that the drop responds to these

perturbations by quickly relaxing into its equilibrium quasi-spherical shape, indicating that 9 mN/m is indeed a significant (albeit generally low) interfacial tension.

RO-TN 651-(PAA/ethanol) system

To perform interfacial tension experiments with the RO-TN 651-(PAA/ethanol) system, we used a stainless steel needle to create a pendant drop of the LC phase inside a bath of a PAA/ethanol solution (10.0% w/w). The experiments were performed at 21∘C. SI Movie 7 shows the production of LC drops inside the polymer solution bath. Even by employing a very low flow rate (0.05 μL/s), a stable drop cannot be formed. Instead, a series of small drops connected by a transient LC jet are produced. The LC jet seems to progressively dissolve in the PAA/ethanol solution, leading to individual RO-TN 651 drops that move towards the bottom of the sample cell due to gravity. Interestingly, drops that come into contact do not coalesce (within the time of the experiment), indicating some extent of stabilization, presumably by PAA chains adsorbing onto the LC-polymer solution interface. In addition, the ejected drops seem to slightly increase in size as they sediment. This suggests that swelling of the LC drop due to ethanol diffusing into its interior might take place. This is further supported by the observation that the drop-solution interface becomes less well-defined, macroscopically, as seen from the decreasing sharpness of the fluid boundary. This qualitative picture is in accordance with the phase behavior of the RO-TN 651-(PAA/ethanol) system (Fig.4) that shows that the two phases are fully miscible at very low ethanol concentrations; the pendant drop experiment described here is analogous to this case, considering that we have droplets with volumes on the order of microliters inside a bath with volume on the order of a few milliliters.

Even if we stop dosing the LC after a first drop is formed, this drop does not remain stable; the LC keeps flowing, even though there is no external pressure imposed on the syringe. The inability to make a stationary drop prevents us from measuring the equilibrium interfacial tension of the RO-TN 651-(PAA/ethanol) system. SI Movie 6 shows the early

stage of the formation of a LC drop, under the optimum conditions we identified (target drop volume 1 μL, flow rate 0.1 μL/s); higher flow rates led to a pronounced ejection of LC drops connected by a LC jet, whereas lower flow rates resulted in the case where the LC drop is not in full contact with the whole orifice of the needle. Under these experimental conditions, a well-defined drop can be formed, which is followed by the formation of a second drop that is however highly non-spherical (due to the broken jet created in its front). Fig. S1 shows snapshots from the formation of the first drop, with $t = 0$ s corresponding to the time where the first image of the shown series was recorded. Clearly, the drop shape analysis cannot give reliable interfacial tension values because the pendant drop is not stationary (pendant drop tensiometry requires a stationary drop, the shape of which is dictated by the balance of surface tension and gravity). Despite this fact, we show in Fig. S1 fits of the drop profile; as can be seen, we can fit reasonably well the shape of the pendant drop recorded in the first two images (i.e., 0 and 0.46 s), while the fitting becomes worse for longer times. Although the calculated, transient interfacial tension values (about 3 mN/m) cannot be trusted absolutely, we get an idea about their order of magnitude; they are very low. This picture of a diminishing interfacial tension between the RO-TN 651 and the PAA/ethanol phases is consistent with what would one expect for an interface formed by two phases consisting of the same chemical constituents, but at different compositions.

(RO-TN 651/ethanol)-(PAA/ethanol) system

To conduct interfacial tension experiments with the (RO-TN 651/ethanol)-(PAA/ethanol) system, we used a stainless steel needle to make a pendant drop of the ethanol-containing LC phase inside a bath of a PAA/ethanol solution (10.0% w/w). The experiments were performed at 21○C. A few problems that prevent the formation of a well-defined, stable pendant drop of LC are shown in SI Movie 8. First, as the LC phase is pushed through the needle, a soft, solid-like phase of irregular shape emerges first. We believe this is highly concentrated (and perhaps gelled) PAA/ethanol solution that was previously left at the

opening of the needle. This can be due to drying of a portion of this solution that was left at the tip of the needle while it is removed from the solution bath in order to be cleaned. Without this cleaning cycle, we are not able to form a pendant drop at all. Furthermore, as more LC is flushed through the needle, an irregularly shaped LC drop emerges which surrounds the solid-like polymer solution phase. At longer times, and with more LC emerging from the orifice, more drops are formed that are initially separated from the first LC drop before they eventually merge to form a large, irregularly-shaped LC drop. The difficulty in merging, in conjunction with the irregular shape of the LC-polymer solution interface (which is nevertheless well-defined), suggests that the LC phase is stabilized by the polymer solution, presumably by PAA chains adsorbing onto the fluid interface. While interesting, these observations clearly show that pendant drop tensiometry is not an appropriate method to measure the interfacial tension of this highly complex system.

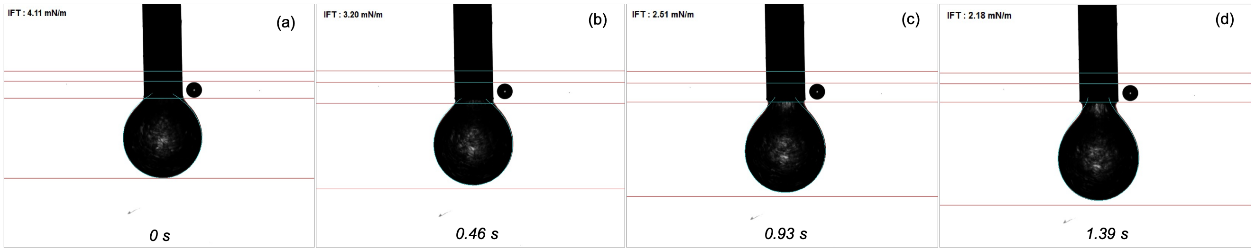

Figure S1: Snapshots of the interfacial tension measurements using pendant drop technique of ROTN 651 to a solution of 10% w/w PAA in anhydrous ethanol. These still frames are extracted from SI movie 6, the outer diameter of the stainless steel needle used for the measurements is 0.51 mm.

Electrospinning Parameters

Table S1 gives the electrospinning parameters used in the experiments.

Table S1: Electrospinning parameters and conditions.

		Relative Humidity(%)	Temperature (∘ C)	Distance (cm)	Voltage (kV)	Flow rate (ml/h)	
Sheath	Core					Sheath	Core
11.5% w/w PAA in water	RO-TN 651	25	22.6	15	9.8	5.1	0.24
10% w/w PAA in anhydrous ethanol	RO-TN 651	24	22.1	15	6.9	19.68	0.24
10% w/w PAA in anhydrous ethanol	10% w/w anhydrous ethanol in RO-TN 651	36	22.6	15	5.4	15.3	0.42

SI Videos

SI Movie 1: Movie of the Taylor cone recorded during electrospinning RO-TN 651 as core and an aqueous solution of 11.5% w/w PAA as sheath. The outer diameter of the spinneret needle is 1.7 mm and the electrospinning parameters and conditions are listed in the Table S1. This movie corresponds to Figure 3.

SI Movie 2: Movie of the Taylor cone recorded during electrospinning RO-TN 651 as the core and a solution of 10% w/w PAA in anhydrous ethanol as sheath. The outer diameter of the spinneret needle is 1.7 mm and the electrospinning parameters and conditions are listed in the Table S1. This movie corresponds to Figure5.

SI Movie 3: Movie of the Taylor cone recorded during electrospinning of 10% w/w anhydrous ethanol in RO-TN 651 core and a solution of 10% w/w PAA in anhydrous as sheath. The outer diameter of the spinneret needle is 1.7 mm and the electrospinning parameters and conditions are listed in the Table S1. This movie corresponds to Figure 6.

SI Movie 4: Movie of the Taylor cone during electrospinning of 10% w/w THF in RO-TN 651 as the core and a 10% w/w SBS in THF, as sheath solution. The outer diameter of the spinneret needle is 1.7 mm. This movie corresponds to the Figure 7.

SI Movie 5: Interfacial tension measurement using pendant drop technique with a drop of RO-TN 651 immersed in a bath of aqueous solution of 11.5%w/w PAA. The flow rate of the pendant drop is 0.5 μL/s and the diameter of the stainless steel needle used is 0.51 mm.

SI Movie 6: Interfacial tension measurement using pendant drop technique with a drop of RO-TN 651 immersed in a bath of 10%w/w PAA solution in anhydrous ethanol. The flow rate of the pendant drop is 0.1 μL/s and the diameter of the stainless steel needle used is 0.51 mm.

SI Movie 7: Interfacial tension measurement using pendant drop technique with a drop of RO-TN 651 immersed in a bath of 10%w/w PAA solution in anhydrous ethanol. The flow rate of the pendant drop is 0.05 μL/s and the diameter of the stainless steel needle used is 0.51 mm.

SI Movie 8: Interfacial tension measurement using pendant drop technique with a drop of 10% w/w anhydrous ethanol in RO-TN 651 immersed in a bath of 10%w/w PAA solution in anhydrous ethanol. The flow rate of the pendant drop is 0.1 μL/s and the diameter of the Teflon needle used is 0.8 mm.
